# Experimental study on Compton camera for boron neutron capture therapy applications

**DOI:** 10.1038/s41598-023-49955-9

**Published:** 2023-12-18

**Authors:** M. Sakai, S. Tamaki, I. Murata, R. K. Parajuli, A. Matsumura, N. Kubo, M. Tashiro

**Affiliations:** 1 Gunma University Heavy Ion Medical Center, 3-39-22 Showa-machi, Maebashi, Gunma 371-8511 Japan; 2 Graduate School of Engineering, Osaka University, Osaka, Japan; 3 Sydney Imaging Core Research Facility, The University of Sydney, Camperdown, NSW 2050 Australia

**Keywords:** Biomedical engineering, Radiotherapy, Cancer, Health care, Oncology, Engineering

## Abstract

Boron neutron capture therapy (BNCT) is a high-dose-intensive radiation therapy that has gained popularity due to advancements in accelerator neutron sources. To determine the dose for BNCT, it is necessary to know the difficult-to-determine boron concentration and neutron fluence. To estimate this dose, we propose a method of measuring the prompt γ-rays (PGs) from the boron neutron capture reaction (BNCR) using a Compton camera. We performed a fundamental experiment to verify basic imaging performance and the ability to discern the PGs from 511 keV annihilation γ-rays. A Si/CdTe Compton camera was used to image the BNCR and showed an energy peak of 478 keV PGs, separate from the annihilation γ-ray peak. The Compton camera could visualize the boron target with low neutron intensity and high boron concentration. This study experimentally confirms the ability of Si/CdTe Compton cameras to image BNCRs.

## Introduction

Boron neutron capture therapy (BNCT) is a promising cancer treatment technique that was first proposed by the American physicist G. L. Locher^1–5^. It exploits the high probability of ^10^B to capture thermal neutrons according to the nuclear reaction ^10^B(n, α)^7^Li^1,6–8^. The accumulation of ^10^B in tumor cells and the external irradiation of neutrons lead to high dose concentrations. Additionally, the products of this reaction (^7^Li nuclides and α particles) have high-linear energy transfer characteristics and high relative biological effectiveness. With the development of accelerator neutron sources, treatment has been available in numerous hospitals^9–14^.

BNCT primarily treats unresectable, locally advanced, and recurrent cancers. Neutrons do not travel in a straight line and are irradiated over a wide area. Therefore, adverse effects must be considered, and the prescribed dose is determined based on the dose for organs at risks^4,15,16^. To evaluate the dose of BNCT, which depends on the neutron flux and boron concentration at the corresponding site, the number of boron neutron capture reactions (BNCRs) must be known.; however, it is difficult to measure both (neutron flux and boron concentration) during the treatment. Currently, the neutron flux is estimated based on simulation calculations and the boron concentration is presumed from the concentration in the blood based on prior tests^17^.

To address this problem, the measurement of prompt γ-rays (PGs) has been proposed^18–20^. The residual nuclide of ^7^Li generated after a BNCR emits PGs at 478 keV with a probability of 94%^20^. If we can quantify the distribution of the PG emission, it will be possible to evaluate the BNCR distribution and the associated BNCT dose.

In the BNCT treatment room, many X- and γ-rays are emitted in addition to the PGs emitted by BNCRs. Among them, annihilation γ-rays (AGs) have an energy of 511 keV, and the energy difference with PGs of 478 keV is only 33 keV (approximately 7%). To distinguish the PGs from the AG with an energy-sensitive detector, high-energy resolution detection is necessary. Moreover, in BNCT treatment rooms, hydrogen and carbon produce 2.2 MeV and 4.4 MeV γ-rays, which are difficult to shield with a conventional gamma camera's mechanical collimator. For example, it requires a thickness of approximately 5 cm of lead to reduce 2.2 MeV gamma rays by less than 10% (even if it don't account for the penetration of scattered rays).

**Figure 1:**
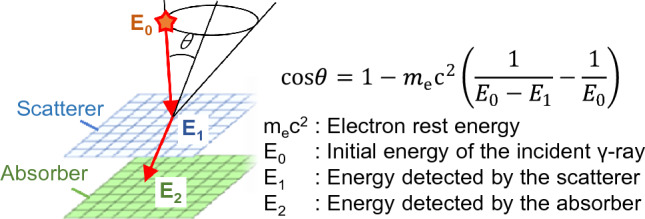
Principle of operation of a Compton camera. An elementary Compton camera consists of two types of position-sensitive subdetectors (scatterer and absorber); it can identify the incidence direction of γ-rays based on the kinematics of Compton scattering.

We have been developing and experimenting with a Compton camera for medical use^21–24^. An elemental Compton camera consists of two position-sensitive detectors, namely the scatterer and absorber. Compton cameras utilize incident γ-rays which are Compton scattered in a scatterer–detector followed by their photo-absorption by an absorber–detector. The scattering angle can be calculated from the detected energies based on the kinematics of Compton scattering (Fig. 1). Because Compton cameras do not require a mechanical collimator, they can detect γ-rays ranging from tens of keV to several MeV even in a high-background environment of high-energy γ-rays^25–28^. A Compton camera with Si and CdTe semiconductor detectors, which has a high-energy resolution, yields high-angular resolution outcomes^29,30^. Several studies have attempted to measure the PGs of the BNCR with Compton cameras. However, most of these have been limited to Monte-Carlo simulations or spectroscopic studies^19,31–35^. In this study, we experimentally confirmed that a Compton camera can image the PGs of BNCRs separately from AGs.

## Methods

### Compton camera

We used a commercial Compton camera (ASTROCAM 7000HS, Mitsubishi Heavy Industries Ltd., Japan), which consisted of eight layers of Si scatterers and four layers of CdTe absorbers^36^. The Compton camera was developed for environmental monitoring following the accident at the Fukushima Daiichi Power Plant. The dimensions of each detector (both scatterers and absorbers) were size = 50 mm (square) and thickness = 0.75 mm. The energy resolution and angular resolution measure (ARM) were 2.2% and 5.4° (full-width-half-maximum) at 662 keV, respectively. It was not modified or specialized for this experiment. Further details were described in a previous study^36^.

### Sources and targets

An ^241^Am^9^Be source was used Ref.^37^. It generated neutrons up to approximately 10 MeV along with 4.4 MeV γ-rays from excited carbon nuclei. The intensities of neutrons and photons were 9.6 × 10^6^ [neutrons/s] and 7.2 × 10^6^ [photons/s], respectively. For the target, 100 g of B_4_C powder (Nilaco, Japan) enclosed in a cylindrical container (diameter = 5 cm and height = 6 cm) were used. The boron target was not isotopically enriched. Thus, the net ^10^B content was 14.5 g.

### Setup

**Figure 2:**
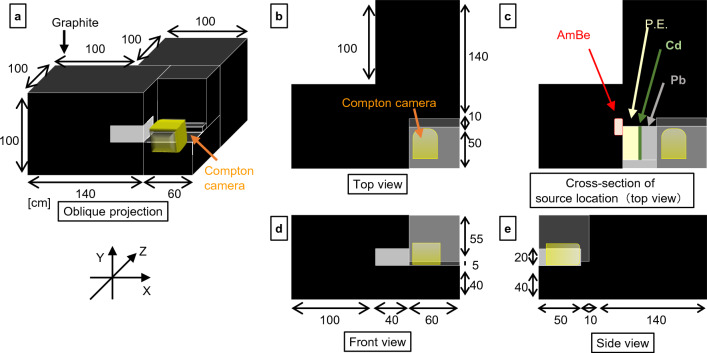
Schematic views of the experimental setup. The oblique view (**a**), the top view (**b**), the cross-sectional top view at the source location (**c**), the frontal view (**d**), and the side view (**e**) of the experimental setup. *P.E*. polyethylene.

**Figure 3:**
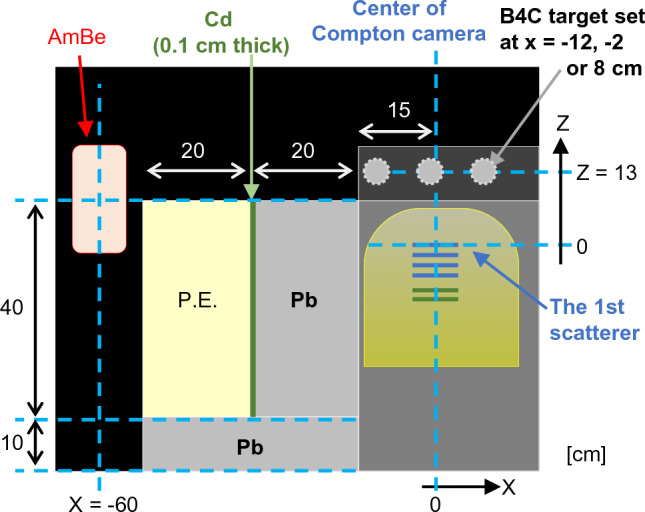
Arrangement of the source, target, and Compton camera in the experimental setup. Enlarged view of Fig. 2c. *P.E*. polyethylene.

Because AmBe produces high-energy neutrons, the neutrons were moderated by graphite to thermal neutrons. The setup (ex. the thickness of graphite, polyethylene, Cd, and lead) was optimized using Monte-Carlo simulations (PHITS 3.24 with JENDL-4.0^38,39^) (data not shown). Figure 2 illustrates the alignment of the Compton camera, sources, and targets from different views, and Fig. 3 shows an enlarged view of the neutron source and camera area.

The subdetectors of the Compton camera were installed vertically. The horizontal and vertical directions were along the X- and the Y-axes, respectively, and the perpendicular direction to the subdetectors was along the Z-axis. The center of the detector was aligned with the origin of the X- and Y-axes, and the position of the first scatterer was set to Z = 0. The lateral sides (± X and ± Y) of the Compton camera were shielded with 0.5 mm thick Cd plates to reduce thermal neutrons.

The AmBe neutron source was placed at X =  − 60 cm, as illustrated in Fig. 3. The fast neutrons produced were moderated by graphite (thickness = 40 cm). Polyethylene, Cd, and lead were used to prevent direct irradiation of the fast neutrons and γ-rays from the AmBe source onto the Compton camera. The polyethylene moderated the neutrons, and the Cd absorbed them. Despite the generation of numerous γ-rays, a thick layer of lead between the Cd and the Compton camera effectively shielded them. To increase the flux of thermal neutrons to the boron target, graphite was also placed around these setups. The B_4_C target was placed at a distance of Z = 13 cm.

To evaluate the effects of water—which generates PGs via the neutron–hydrogen reaction (2.2 MeV)—On the images, imaging was also performed with 20 g of B_4_C powder (2.9 g of ^10^B) sealed in a plastic container (size of X × Y × Z = 0.7 × 9.6 × 2.2 cm^3^), which was placed in a 10 cm cubic water tank. The water tank was set up along the x, y, and z axes, and its center was at X = 0, Y = 0, and Z = 13 cm. The B_4_C target was set at X =  − 2, Y = 0, and Z = 13 cm in the water tank.

### Imaging conditions

The B_4_C targets were set at X =  − 12, − 2, or 8 cm, and each was measured for 2 h. To evaluate the effects of γ-ray scattering by the target on the reconstructed images, measurements were also performed with a graphite block of the same size as that of the B_4_C target set at X =  − 2 cm.

### Data processing

From the Compton camera, only two-hit events with Si and CdTe were extracted; single interactions were measured by Si and CdTe. Data with three or more interactions and data associated with interaction occurrences with Si–Si or CdTe-CdTe were not included.

Among the extracted two-hit data, we selected data that met the following criteria to reconstruct images. The energy window was set to exclude data from the peak of 511 keV.

1. Sum of energies in the range of 468–488 keV,
2. The energy detected by a Si scatterer (E1) < 200 keV (to remove backward events and noise),
3. E1 < 20 keV or E1 > 35 keV if the data were detected in the last scatterer and the first absorber (to exclude coincidence measurements with characteristics of CdTe X-rays).

Images were reconstructed with a back-projection reconstruction and improved with maximum-likelihood expectation–maximization imaging techniques^29^. The initial energy and the energy measured at the scatterer were used to calculate the back-projections; the initial energy of PGs from BNCR was assumed to be 478 keV (i.e. the Doppler effect of BNCR was neglected in the reconstruction calculations^40^).

### Background reduction

In this experiment, noise data from many γ-rays and printed circuit boards (PCB) were measured. Thus, to improve the image quality and discriminate the artifacts by removing the effects of the noise data, we subtracted the image reconstructed with the graphite target (SIwG) or the image without the target (SIwoT) from each image in which the target was present (on a pixel-by-pixel basis). To evaluate the effect of the subtraction, we calculated the PVR between the areas with B_4_C targets and the other areas. In the PVR calculation, it was calculated as 0, if the pixel value was negative.

## Results

### Distributions of neutrons and photons

**Figure 4:**
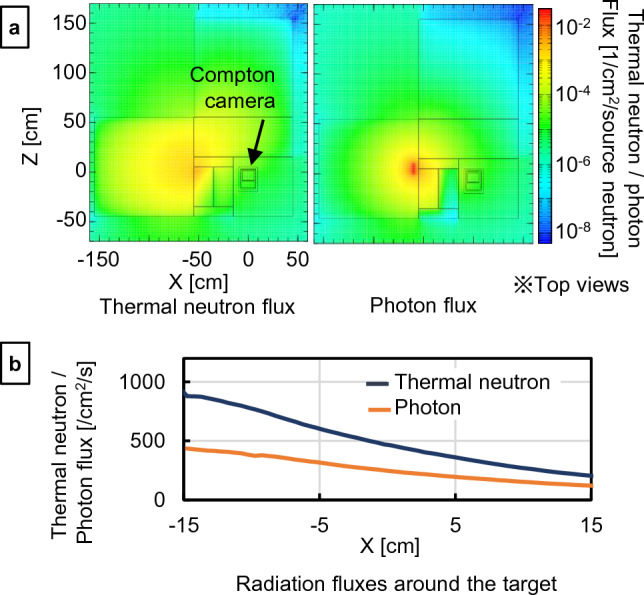
Flux distributions of thermal neutrons and photons calculated by PHITS 3.24. (**a**) Two-dimensional distributions of thermal neutrons and photons at the slice Y = 0. (**b**) Variations of the thermal neutron and photon fluxes around the target (Y = 0 and Z = 13 [cm]) along the X direction.

The distributions of neutrons and photons in the experimental setup were calculated using PHITS (Fig. 4). In this flux-distribution calculation, there was no boron target. In the region where the B_4_C target was installed in the experiment (Y = 0 and Z = 13 cm), the thermal neutron and γ-ray fluxes decreased along the X-axis (Fig. 4b). The thermal neutron fluence was calculated to be approximately 2.7 × 10^6^ n/cm^2^ based on the 2 h measurement at X = 0.

### Energy spectrum measured by Compton camera

**Figure 5:**
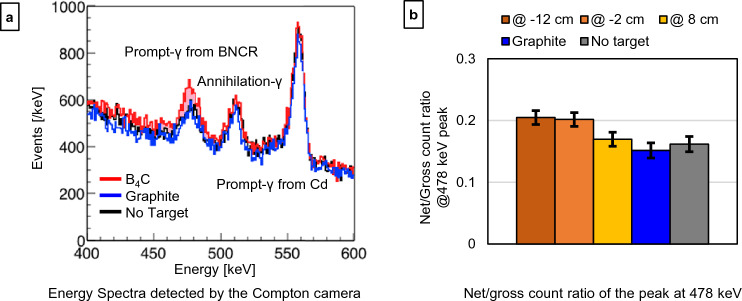
PG signals in the energy spectra. Energy spectra detected by the Compton camera (**a**) and the net/gross count ratio of the peak at 478 keV (**b**). In (**a**), the colored lines show the spectra with the B_4_C target at − 2 cm (red line), the graphite target at − 2 cm (blue line), and the spectrum obtained without a target (black line). The error bars in (**b**) show the standard deviations.

The energy spectra detected by the Compton camera with the B_4_C target and the graphite target at X =  − 2 cm were measured and compared with those obtained without a target (Fig. 5a). The PGs of 478 keV and AGs (at 511 keV) were observed separately. Even in the absence of a target, a peak at approximately 478 keV was observed. (The presence of boron in the PCB could contribute to this peak, as discussed in the following section.) To confirm that the peak of 478 keV contains the PG signals from the B_4_C target, the net/gross count ratio of the peaks was calculated (Fig. 5b). The number of background events was estimated by linear fitting to the 450–463 keV and 493–499 keV data in each spectrum. The results demonstrated that the ratio was higher when the B_4_C target was placed at X =  − 12 cm or − 2 cm compared with its values in other conditions.

### Reconstructed images

**Figure 6:**
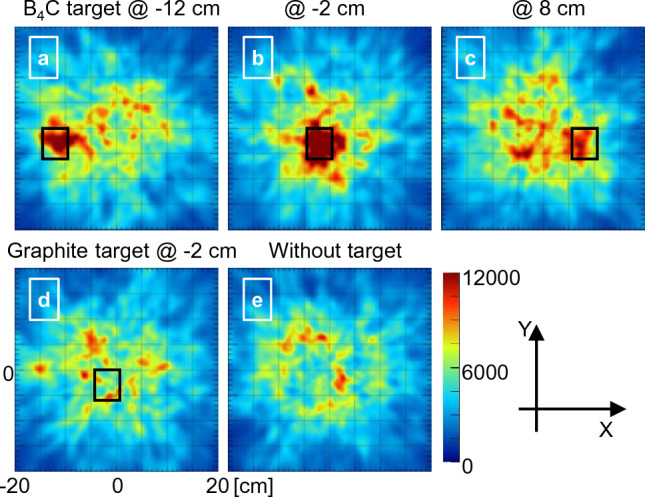
Compton images reconstructed using the 478 keV peaks. Compton images with the B_4_C target placed at (**a**) X =  − 12, (**b**) − 2, and (**c**) 8 cm. Images obtained (**d**) with the graphite target at − 2 cm and (**e**) without a target. The color map is the same for all images. The black square in the images indicates the target position. The scale of all images is the same.

The number of events used for image reconstruction were 5929, 6267, 5814, 5503, and 5261 for the conditions with the B_4_C target at X = − 12, − 2, and 8 cm, with a graphite target, and without a target, respectively. Figure 6 illustrates the reconstructed Compton image wherein a high pixel value region is observed at the location of the B_4_C target when placed at X =  − 12 cm or − 2 cm. However, the signal was not distinguishable from background fluctuations when the B_4_C target was at X = 8 cm.

No differences were observed when the graphite target was in place at − 2 cm compared with the outcome obtained in the absence of the target. In addition, no images correlated with the target position when other energy windows were used (Supplementary Fig. S1).

### Background subtraction

**Figure 7:**
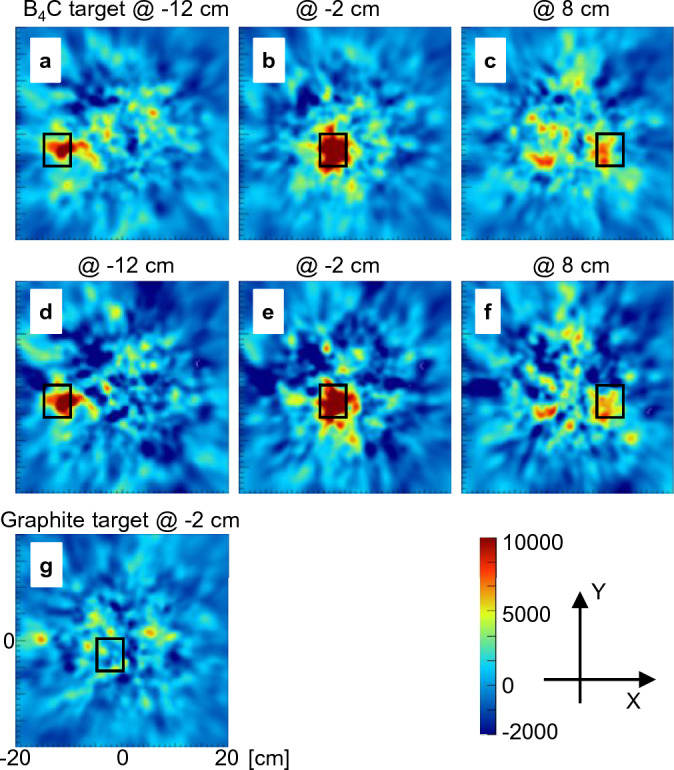
Improved Compton (subtracted) images. They were obtained by subtracting the image without a target from the Compton images acquired with the B_4_C target at positions (**a**) X =  − 12, (**b**) − 2, or (**c**) 8 cm. Alternatively, the image with a graphite target was subtracted from the images with the B_4_C target at positions (**d**) X =  − 12, (**e**) − 2, or (**f**) 8 cm. Additionally, (**g**) was obtained by subtracting the image without a target from the images with the graphite target at 8 cm. The color map is the same for all images. The black square in the images indicates the target position. The scale of all images is the same.

Figure 7 shows the improved Compton images (after the background image was subtracted). The contrast improved compared with the original images. The ratios of the mean pixel values (PVR) for the images with the B_4_C target at X =  − 12, − 2, and 8 were 3.5, 5.1, and 3.0, respectively, in the original image. However, these ratios increased to 6.3, 11.2, and 4.7 in SIwG and to 11.6, 16.6, at 7.0 in SIwoT.

### Imaging of B_4_C placed underwater

**Figure. 8:**
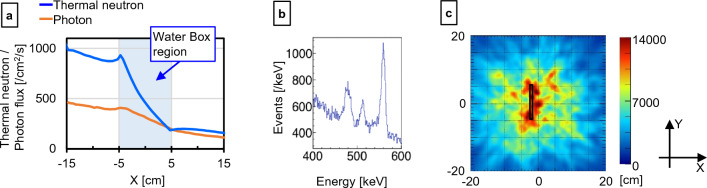
Underwater target Compton camera data. Flux distributions of thermal neutron and photon (**a**), energy spectrum detected by the Compton camera (**b**), and imaging result (**c**) from the experiment with the water tank. The black rectangular block represents the B_4_C target position.

Figure 8a shows the neutron and photon fluxes when a water tank is installed in front of the Compton camera. Additionally, Fig. 8b shows the energy spectrum detected by the Compton camera, and Fig. 8c shows the imaging results of the B_4_C target (black rectangular block in Fig. 8c) in the water tank. Even when the target was inside the water, the target can be observed in the reconstructed image.

## Discussion

This study was conducted to demonstrate the feasibility of the Compton camera for BNCR imaging in BNCT. We were able to observe the peak of PGs of 478 keV separately from AGs in the obtained energy spectra with the Compton camera (Fig. 5). It was confirmed that the energy resolution of the Compton camera was sufficient to discriminate between the PG and AGs. The absorber of CdTe may not be suitable for installation in a neutron field because of the Cd’s large cross-section with thermal neutrons. When Cd absorbs neutrons, the decay with 558 keV γ-rays produces noise in the detector. If an alternative detector is used, it must possess comparable energy resolution and have the same level of energy resolution. Certain published studies stated that sufficient energy resolution can be obtained when Ge and TlBr are used as absorbers^31,32^.

Even without the B_4_C target, the PG peak was observed (Fig. 5) owing to the boron atoms in the PCB)^41^. Borosilicate glass, commonly used in PCBs, contains a substantial amount of boron^42^. While it is challenging to pinpoint the distribution of boron within the Compton camera without disassembly, observations by a NaI scintillator showed that 478 keV γ-rays were emitted from the Compton camera set in the neutron field (Supplementary Fig. S2). However, when the B_4_C target was installed at Z =  − 12 or − 2 cm, higher intensity peaks were observed compared with those obtained when there was no B_4_C target. Thus, we believe that the BNCR signal generated within the B_4_C target was observed. When the target was at Z = 8 cm, the decrease in neutron flux to the target and the increase in distance from the target to the Compton camera would reduce the signal intensities, and they buried the signal in noise of scattered γ-rays and the PGs from the PCB.

To reduce the γ-rays from the Cd and boron inside the Compton camera, it's essential to minimize the number of incident neutrons on the Compton camera. Shielding the lateral side of the Compton camera with Cd plates had limited impact, as the height of the 478 keV peak remained nearly unchanged, even when measurements were taken without shielding (the 558 keV peak was smaller). This is likely due to the high number of neutrons entering the Compton camera from the front side and the moderation of epi-thermal neutrons not shielded by the Cd. Therefore, designing a Compton camera for BNCR imaging should incorporate neutron shielding.

Reconstructed images using the data visualized the B_4_C target when it was placed at X =  − 12 or − 2 cm (Fig. 6); when the target was placed at X = 8 cm, the signal level was nearly the same as that of background noise. When graphite targets were placed or when other energy windows were used, the targets could not be visualized (Fig. 6d, Supplementary Fig. S1). Therefore, these images are not due to artifacts or incidental findings caused by the setup and/or the Compton camera itself. This was also supported by the fact that the image quality was significantly improved by the subtraction of background components (difference of subtracted images with and without the target (SIwoT) and with graphite (SIwG)). The SIwoT image cannot exclude the effects of γ-rays scattered by the target, because the image without a target does not include the data with the gamma-rays scattered by a target. In addition, the B_4_C target absorbs thermal neutrons, which may reduce the effects of PGs generated by the PCB. However, the SIwoT and SIwoG images are almost identical, and their effects are extremely small. Assuming that the data measured with the graphite target represents the background (BG) component of imaging the B_4_C target, the difference is likely the PG signal from the B_4_C target. The amount of that signal correlates with the amount of BNCR and is important for quantitative measurements (Supplementary Fig. S3). However, the statistical error is significant in this study, and a more precise measurement is required for quantitative evaluation.

Finally, imaging was performed with the B_4_C target submerged in water (Fig. 8). Due to the reaction of neutrons with water and scattering by water, more γ-rays enter the Compton camera. Even in these conditions, the Compton image was able to show the location of the B_4_C target. Notably, 2.2 MeV γ-rays generated by the interaction between water and neutrons are challenging to shield with a mechanical collimator. However, in the case of the Si/CdTe Compton camera, the probability of 2.2 MeV γ-rays being scattered by the Si scatterer is very low, and the probability of the scattered γ-rays being scattered by the CdTe, resulting in a total deposition energy of 478 keV, is even lower (less than 10% of the probability of a 478 keV PG ray being measured correctly). The high energy resolution reduces random noise, and the impact on the reconstructed image is minimal because false data randomly reconstructs Compton cones independent of the original source position. Therefore, the crosstalk effect is limited in Compton cameras. This could be one reason why imaging was possible even in the presence of high-energy γ-rays^43^. This suggests that BNCR can be imaged even in water (or inside the body) if there is a sufficient amount of BNCR.

The intensity of the AmBe neutron source used in this study was much smaller than that of a clinical source. In addition, it also produced high-energy neutrons and γ-rays^37^. Therefore, only a few thermal neutrons reached the experimental field with neutron moderation and shielding against direct exposure to high-energy neutrons and γ-rays (Fig. 4). The flux of the moderated thermal neutrons was approximately 5–6 orders lower in magnitude compared with that of the therapeutic and numerous γ-rays contaminated it. These deteriorated the measurements of the PGs condition^44–47^ and numerous γ-rays contaminated it. These deteriorated the measurements of the PGs.

Conversely, B_4_C powder was used in this study. This had a ^10^B concentration of 14.5% (weight percentage), which is considerably higher than clinical conditions (~ 80 parts per million (ppm)))^47–50^. Imaging a lower concentration of ^10^B is expected in future experiments. Even though the amount of boron used in this study was large, all thermal neutrons reacted with ^10^B in a small volume of the target surface (less than 0.1 mm of thickness), and the amount of ^10^B contained in the volume was estimated to be significantly less than 1 g ^10^B (because the cross-section of boron was large, the probability of thermal neutrons penetrating a 0.1 mm thick B_4_C was less than 1%, and most thermal neutrons could not penetrate into the target). Assuming that the clinical conditions of a neutron flux of 1 × 10^9^ n/cm^2^/s, an intratumor ^10^B concentration of 80 ppm, and a tumor volume of 100 cm^3^, the amount of BNCR generated during the experiments in this study (1 × 10^3^ n/cm^2^/s, 1 g ^10^B, and 7200 s) was comparable to that produced during treatments which lasted 1 s. Under high thermal-neutron flux conditions, the noise from the PBC is also expected to increase dramatically. It would be better to use boron-free PCBs^51–53^. The increase in both signal and noise will also increase the dead time. Medical applications require the development of high-speed response Compton cameras, which is expected to be improved by improving application specific integrated circuits.

The detection efficiencies of the Compton camera for 356 keV and 511 keV point sources placed at a distance of 100 mm were 2.7 × 10^–6^ and 1.5 × 10^–6^, respectively, in a previous study (data not shown). Thus, the detection efficiency for 478 keV can be estimated to be ~ 2 × 10^–6^. This detection efficiency is higher than that of a multihole collimator-based detector (Anger camera^54,55^). However, the image reconstruction of the Compton camera is complex and requires more data than the Anger camera to obtain a comparable image quality. Conversely, the effect of high-energy γ-rays may be small^56^; thus, comparisons should take into account the signal-to-noise ratio and other factors. Therefore, it is not possible to determine which method is superior, at this point. The angular resolution could be also estimated to be in the range of 5–6°^23,57,58^ (it is the slope that shifts approximately 8.7–10.5 mm at 10 cm ahead) if the effect of Doppler broadening is small. If the required spatial resolution is set at 10 mm^59^, it can be achieved with simple modifications, e.g., by changing the distance between detectors^60^. Although adjusting the distance between detectors reduces detection efficiency, it is feasible because the efficiency is very high at present. The current imaging does not provide sufficient image quality to evaluate spatial resolution, concentration resolution, and provide quantitative estimates. It is required to evaluate the imaging capability with high-neutron flux and low-γ-rays contamination, such as those encountered in a treatment room.

For clinical applications, three-dimensional measurements are required to image the distribution of the reaction in a patient's body. It is also necessary to develop algorithms to calculate doses from reconstructed images (quantitative method). These studies are being conducted in the field of nuclear medicine applications^22,28,61–67^. Although there are numerous considerations to be taken into account for clinical applications, this study successfully visualized BNCR and experimentally demonstrated the potential of the Compton camera.

## Conclusion

In summary, this study verified the possibility of Compton imaging of 478 keV PGs generated in BNCR. The results demonstrated that the Si/CdTe Compton camera can measure PGs discriminated from the AGs of 511 keV and image the position of the boron target. Although additional studies are necessary because the conditions in this study were very different from the treatment conditions, we were able to demonstrate the potential application of the Compton camera for use in BNCT.

### Supplementary Information

Supplementary Figures.

## Data Availability

The datasets used and/or analyzed during the current study available from the corresponding author on reasonable request.
