# Author Correction: Experimental study on Compton camera for boron neutron capture therapy applications

**DOI:** 10.1038/s41598-024-53559-2

**Published:** 2024-02-07

**Authors:** M. Sakai, S. Tamaki, I. Murata, R. K. Parajuli, A. Matsumura, N. Kubo, M. Tashiro

**Affiliations:** 1 Gunma University Heavy Ion Medical Center, 3-39-22 Showa-Machi, Maebashi, Gunma 371-8511 Japan; 2 Graduate School of Engineering, Osaka University, Osaka, Japan; 3 Sydney Imaging Core Research Facility, The University of Sydney, Camperdown, NSW 2050 Australia

Correction to: *Scientific Reports* 10.1038/s41598-023-49955-9, published online 18 December 2023

The original version of this Article contained an error in the name of R. K. Parajuli, which was incorrectly given as R. K. Parajul.

The original Article has been corrected.

