# Supplementary material for: Experimental study on Compton camera for boron neutron capture therapy applications
Source: Sci Rep. 2023 Dec 18;13:22883. doi: 10.1038/s41598-023-49955-9 (PMC10739814; doi:10.1038/s41598-023-49955-9)
Supplement: Supplementary file 1 — Supplementary Figures. [file 41598_2023_49955_MOESM1_ESM.docx]

Supporting Materials


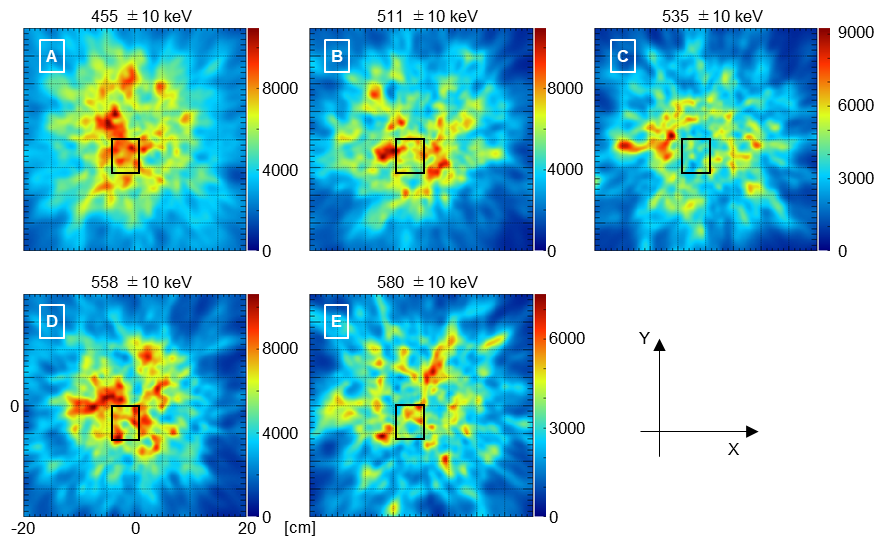


**Supplementary Figure S1**. Reconstructed Compton images with the B_4_C target set at x = -2 cm. The black rectangular block represents the B_4_C target position. The energy window used to reconstruct was set at (A) 455 ± 10 keV, (B) 511 ± 10 keV (peak of annihilation γ-rays), (C) 535 ± 10 keV, (D) 558 ± 10 keV (peak of prompt γ-rays of Cd), and (E) 580 ± 10 keV. The scale of all images is the same.


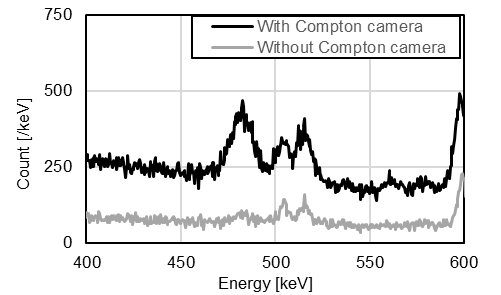


**Supplementary Figure S2.** Energy spectra detected by NaI scintillators. When the Compton camera was set in front of the NaI scintillators (black line), the peak of prompt γ-rays from the Boron neutron capture reaction was observed at 478 keV.


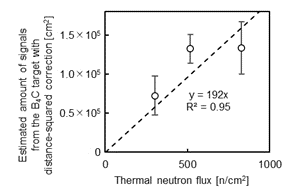


Supplementary Figure S3. The vertical axis expresses the values which calculated by subtracting the number of events in the measurement with the graphite target from the number of events in the measurement with the B_4_C target and then correcting for the square of the distance from the Compton camera to the B_4_C target.
